# Modeling and analysis for the transmission dynamics of cotton leaf curl virus using fractional order derivatives

**DOI:** 10.1016/j.heliyon.2023.e16877

**Published:** 2023-06-05

**Authors:** Abayneh Kebede Fantaye, Zerihun Kinfe Birhanu

**Affiliations:** a Debre Tabor University, Department of Mathematics, Ethiopia; b Hawassa University, Department of Mathematics, Ethiopia

**Keywords:** Modeling, Cotton leaf curl viruses, Hyers-Ulam stability, Fractional order, Numerical simulation

## Abstract

In this study, we examine the Atangana Baleanu Caputo fractional order for the transmission dynamics of the Cotton Leaf Curl Virus disease. The model took into account both cotton plants and vector populations. The existence and uniqueness, positivity and boundedness of the solution to the model, as well as other fundamental concepts, were examined. Additionally, the Ulam-Hyres condition stability of the suggested model was demonstrated using functional techniques. Using the Adams-Bashforth method, the numerical solution for our suggested model was computed. The numerical result shows that the disease spreads more slowly as the fractional order decreases from 1.00 to 0.72.

## 1 Introduction

Cotton is special fiber crop in many applications. It is renowned for its adaptability, performance, appearance, typical convenience, and, particularly, various applications in things like astronaut in-flight time blazers, shelters, sleeping bags, and anything else kinds of clothing [1]. It has been grown and used in Ethiopia since ancient times. Cotton is the primary crop in the globe for producing protein, fiber, and energy [2]. Pricing of Ethiopian cotton has started at the Textile Industry Development Institute [3]. Vectors may damage these cotton crops. Crop productivity is low as a result of these conditions. The plant's leaf is most likely to be impacted by infections, which can be fatal or harmful [4]. Cotton Leaf Curl Viruses (CLCuV) disease is a significant barrier to cotton growth. CLCuV is brought on by the Begomovirus genus, a serious threat to the cotton crop that is spread by whiteflies [5]. Usually 2–3 weeks after B. tabaci immunization, cotton symptoms start to manifest. The main method of control is the use of insecticides against the insect vector of the CLCuV, Bemisia tabaci [6].

Currently, there is a lot of discussion about an eco-epidemiological mathematical model of crop disease. In [7], the author established a model of alone jersey knitwear made of plain bleached cotton that could be used to forecast fabric attributes and identify geometric relationships prior to fabrication. A model developed by [8] reveals the differential equations of prey-predator, crop-pest, and migrating effect interactions. A model of fiber coloring in structures on 3 different scales—micro, meso, and macro—was created by the authors in [9]. The author [10] offered mathematical and statistical modeling and statistical models for decision-making, including residual plot analysis and ANOVA-based comparative tests. The study in [11] introduced a single boundary value parabolic problem for computing a temperature within drum dryers for the cotton fiber and atmosphere elements.

For India's cotton fabric region, manufacturing, and output data from 1980 to 2013, Sundar Rajan and Palanivel [12] offered six non-linear growth models: monomolecular, logistics, Gompertz, Richards, quadratic, and reciprocal growth. To forecast the size of the leaves of a cotton crop over consumption in farming systems, a multiple regression model was developed in [13]. To increase the use of water and identify the ideal soil conditioner yield levels. [14] looked into the leaf area index (LAI) algorithms and the connections among LAI, moisture content, and yield over cotton produced in Korla, Xinjiang, China, using three different nutrients for the soil. [15] explored the connection between silverleaf whitefly populations, environmental variables, and CLCuV incidence in Pakistan's farming mechanism. The relationship between the Pakistani cotton leaf curl virus outbreak and weather variables was simply modeled in [16]. In [17], the author created a model for CLCuV by dividing the total population into populations of cotton plants and vectors. Both populations include subgroups that are infected and susceptible.

Fractional calculus is crucial to create more accurate results and to better understand how the memory effect influences epidemiological models. Simply said, it is more adaptive than classical calculus because of inherited traits and memory descriptions [18]. Basically, fractional derivatives are distinguished from one another by the several kernels that can be chosen to suit the requirements of diverse applications. The main distinction between the Caputo fractional derivative [19], Caputo-Fabrizio derivative [20], and Atangana-Baleanu fractional derivative [21] is that while the Caputo derivative is characterized by a power law, the Caputo-Fabrizio derivative is characterized by a decaying law, and the Atangana-Baleanu derivative is characterized by a Mittag-Leffler law.

Many researchers have looked into the possibility that memory effect can be incorporated into fractional calculus [22], [23], [24]. This memory uses information from the past and present to make predictions. Because of this, it differs from integer derivatives. Numerous practical applications of fractional calculus have led to the development of both analytical and numerical approaches to solving problems [25], [26], [27], [28], [29], [30], [31], [32] and [33]. In fractional derivatives, several studies have recently been developed. One of the best operators is the Atangana Baleanu-Caputo (ABC) operator [34]. An expanded Mittag-Leffler function with a nonsingular and nonlocal kernel serves as the foundation for this operator.

However, there haven't been any studies done to use a fractional order derivative to model the dynamics of CLCuV transmission. In this study, we extend the ABC fractional order derivatives for CLCuV diseases from the model created by [17] to other diseases. The remaining sections of the essay are organized as follows: we complete a few foundational ideas for the Atangana-Baleanu fractional operator in part two. The model is developed in part three. Part four establishes the model's existence and uniqueness, as well as positivity and boundedness for the model. The model's stability analysis using Hyers-Ulam stability is covered in part five. In parts six and seven, we use the ABC for numerical scheme and MATLAB software for simulation to the CLCuV model. In part eight, conclusions are provided.

## 2 Preliminaries

Now, let us start by reviewing the basic definitions of Atangana-Baleanu Caputo (ABC) fractional operators.

Definition 1 Suppose that $f\in C^{1}(\alpha ,\beta ),\alpha <\beta$, be a function, and let υ∈[0,1]. In Caputo type of order *υ*, the ABC fractional derivative is defined by [35], [36], [37]

DtυαABCf(t)=G(υ)1−υ∫αtdfdρEυ[−υ1−υ(t−ρ)υ]dρ,

where G(υ) is the normalization function given by $G(\upsilon )=1-\upsilon +\frac{\upsilon }{\Gamma (\upsilon )}$, with G(0)=G(1)=1, and the Mittag-Leffler function $E_{\upsilon }(z)$ with the set of the complex number C is represented by

$$E_{\upsilon }(z)=\sum_{b=0}^{\infty }\frac{z^{b}}{\Gamma (1+\upsilon b)},\upsilon ,b\in C,R(\upsilon )>0.$$

Definition 2 The ABC fractional integral for $f\in C^{1}(\alpha ,\beta )$ is given by [36], [37]

ItυαABf(t)=1−υG(υ)f(t)+υG(υ)Γ(υ)∫αtf(ρ)(t−ρ)υ−1dρ.

Lemma 1 **[38]**

*The ABC fractional derivative as well as integral of* $f\in C^{1}(\alpha ,\beta )$ *fulfills the Newton-Leibniz equality*

ItυαAB(DtυαABCf(t))=f(t)−f(α).

Lemma 2 **[39]**

*For two functions* $f,g\in K^{1}(\alpha ,\beta ),\alpha <\beta$ *, the following inequality holds for the AB fractional derivative:*

$$\|{}_{\alpha }^{ABC}D_{t}^{\upsilon }f(t){-}_{\alpha }^{ABC}D_{t}^{\upsilon }g(t)\|\leq K\|f(t)-g(t)\|.$$

## 3 Model formulation

The CLCuV model was split up into cotton plant and vector population for this study. The total cotton plants ($N_{c}(t))$ as a whole is divided into susceptible and infected subcategories. Cotton that is prone to infection is designated as $I_{c}$ and susceptible cotton as $S_{c}$. This is defined as: $N_{c}(t)=S_{c}+I_{c}$. Within the entire vector population $\left(N_{v}(t)\right)$, there are susceptible and infected vectors subclasses. The vectors $X_{v}$ and $Y_{v}$ stand for the susceptible vector and the infected vector, respectively. This is defined as: $N_{v}(t)=X_{v}+Y_{v}$. The rate of hiring of vulnerable vector $m_{2}$ and the transition to infected vectors $\left(Y_{v}\right)$ with $\alpha_{2}$ rate after consuming diseased plants or cotton were both considered in the model. When infected vectors $\left(Y_{v}\right)$ eat susceptible cotton $\left(S_{c}\right)$, the diseases propagate to cotton at a rate of $\alpha_{1}$. The susceptible cotton $\left(S_{c}\right)$ also replanted at a rate of $m_{1}$ then the cotton was infected with the diseases. Once infected, cotton never recovers and yields either nothing at all or very little. To regulate the illness, the parameter $\beta_{1}$ is the induced death rate and $\beta_{2}$ is the elimination rate of infected cotton plants from uninfected cotton plants. Further more, *d* and *ψ* are natural death rate of cotton plants and vector population respectively.

**Figure 1:**
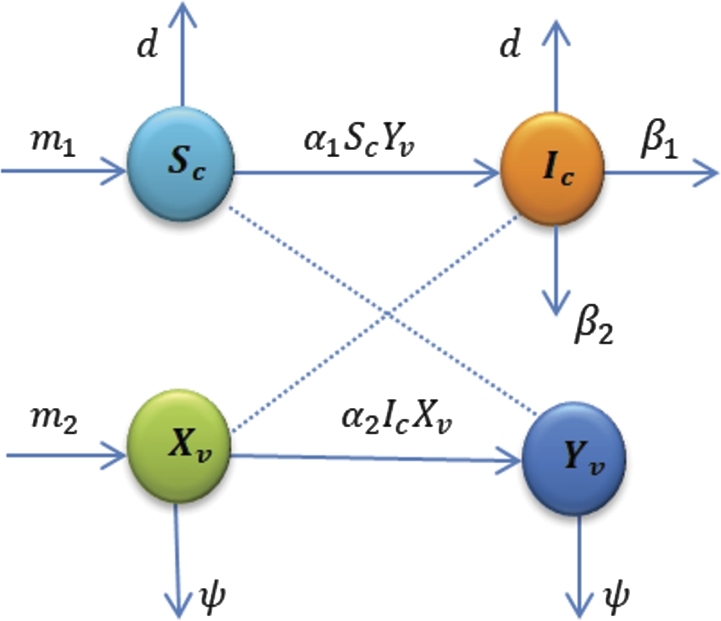
Flow chart of the model.

Based on the assumptions along with flowchart in Fig. 1, the following governing equations are provided:

(1) $$\frac{dS_{c}}{dt}=m_{1}-\alpha_{1}S_{c}Y_{v}-dS_{c},\frac{dI_{c}}{dt}=\alpha_{1}S_{c}Y_{v}-(d+\beta_{1}+\beta_{2})I_{c},\frac{dX_{v}}{dt}=m_{2}-\alpha_{2}I_{c}X_{v}-\psi X_{v},\frac{dY_{v}}{dt}=\alpha_{2}I_{c}X_{v}-\psi Y_{v}.$$

The AB derivative can be derived by changing the $\frac{d}{dt}$ in the system (1) to Dtυ0ABC, which is defined by the differential equation system given in equation (2).

(2) Dtυ0ABCSc(t)=F1(t,Sc),Dtυ0ABCIc(t)=F2(t,Ic),Dtυ0ABCXv(t)=F3(t,Xv),Dtυ0ABCYv(t)=F4(t,Yv),

where the kernels are provided by

$$F_{1}(t,S_{c})=m_{1}-\alpha_{1}S_{c}Y_{v}-dS_{c},F_{2}(t,I_{c})=\alpha_{1}S_{c}Y_{v}-(d+\beta_{1}+\beta_{2})I_{c},F_{3}(t,X_{v})=m_{2}-\alpha_{2}I_{c}X_{v}-\psi X_{v},F_{4}(t,Y_{v})=\alpha_{2}I_{c}X_{v}-\psi Y_{v},$$

with initial conditions

(3) $$S_{c}(0)>0,I_{c}(0)\geq 0,X_{v}(0)\geq 0,Y_{v}(0)\geq 0.$$

Lemma 3 **[40]**

*Suppose that*f(x)∈C[α,β]*, and*Dtυ0ABCf(x)∈C[α,β]*when*0<υ≤1*. Then, we obtain*$f(x)=f(\alpha )+\frac{1}{\Gamma (\upsilon )}$Dtυ0ABCf(σ)(x−σ)n*, when*0≤σ≤x,∀x∈(α,β]*.*

Here, using this Generalized mean value theorem, if f(x)∈[0,β],Dtυ0ABCf(x)∈C[0,β] and Dtυ0ABCf(x)≥0,∀x∈(0,β] when 0≤υ≤1, then the function f(x) is increasing, and if Dtυ0ABCf(x)≤0,∀x∈(0,β] then the function g(x) is decreasing ∀x∈(0,β].

Theorem 1 *The eco-epidemiologically feasible region of AB model*(2)*is given by:*

(4) $$\Omega =\Omega_{c}\times \Omega_{v}=\left\{(S_{c},I_{c},X_{v},Y_{v})\in R_{+}^{4}:N_{c}\leq \frac{m_{1}}{d},N_{v}\leq \frac{m_{2}}{\psi }\right\}.$$

Using Lemma 3, we can demonstrate that the set Ω is positively invariant.

(5) Dtυ0ABCSc|Sc=0=m1≥0,Dtυ0ABCIc|Ic=0=α1ScYv≥0,Dtυ0ABCXv|Xv=0=m2≥0,Dtυ0ABCYv|Yv=0=α2IcXv≥0.

Due to (5), all of (5)'s solutions are nonnegative and remain in $R_{+}^{4}$, making the set Ω established in (4) positively invariant for the equation system (2). Finally, given that all of the parameters are positive, we move on to the next step, the boundedness of the fractional model's solutions (2), by adding up all of the model's equations for cotton and the vector population, which results in:

(6) Dtυ0ABCNc=m1−dNc−(β1+β2)Ic≤m1−dNc,

(7) Dtυ0ABCNv=m2−ψNv.

Using the Laplace transform to the equation (6) gives

Ł(Dtυ0ABCNc+dNc)≤Ł(m1)Ł(Nc)((1−ρ)sυ−ρυ1−υ)−sυ−1Nc(0)≤1−υG(υ)(sυ+υ1−υ)m1s,Ł(Nc)≤(1−ρυ(1−ρ)(1−υ)s−ρ)−1[1−υ(1−ρ)G(υ)(1+υ1−υs−υ)m1s+Nc(0)1(1−ρ)s],

where $\rho =\frac{-d(1-\upsilon )}{G(\upsilon )}$. Using the work which is done by [41] and applying the inverse transform, the solution will be provided by:

$$N_{c}\leq \frac{m_{1}}{d}-\frac{m_{1}}{\left(d(1-\rho )\right)}\frac{d}{dt}\int_{0}^{t}E_{\rho }\left(\frac{\rho \upsilon }{\left(1-\rho )(1-\upsilon \right)}{\left(t-x\right)}^{\upsilon }dx\right)+\frac{1}{1-\rho }E_{\upsilon }\left(\frac{\rho \upsilon }{\left(1-\rho )(1-\upsilon \right)}t^{\upsilon }\right)N_{c}(0),$$

where $E_{a,b}$ refers to the Mittag-Leffler function. Given that the Mittag-Leffler function exhibits asymptotic behavior

$$E_{a,b}(z)\approx \sum_{r=1}^{\beta }z^{-r}/\Gamma (b-ar)+O(|z{|}^{-1-\beta }),|z|\to \infty ,\frac{a\pi }{2}<|\arg(z)|\leq \pi ,$$

it is not difficult to observe that $N_{c}(t)\to \frac{m_{1}}{d}$ for *t* goes to ∞. Also, applying Laplace transform to the equation (6) gives

Ł(Dtυ0ABCNc+υNc)=Ł(m2)

Similarly by using the same procedures to equation (7) we obtain $N_{v}(t)\to \frac{m_{2}}{\upsilon }$ for *t* goes to ∞. Thus, (4) is the biologically feasible region of system of equation (2).

## 4 Existence and uniqueness of solutions

This part investigates the existence and uniqueness of the solutions to the fractional-order model (2). We apply the well-known Banach fixed point theorem to show the existence of the solution to model (2), a detailed analysis of fixed points and contractions, using [42] applying and the references within it. We now go through the steps to show that the solution exists and is distinct. Model (2) yields the following when the AB fractional integral is used.

(8) $$S_{c}(t)-S_{c}(0)=\frac{1-\upsilon }{\Gamma (\sigma )}F_{1}(t,S_{c})+\frac{\upsilon }{G(\upsilon )\Gamma (\upsilon )}\int_{0}^{t}F_{1}(\rho ,S_{c}){\left(t-\rho \right)}^{\upsilon -1}d\rho ,I_{c}(t)-I_{c}(0)=\frac{1-\upsilon }{\Gamma (\upsilon )}F_{2}(t,I_{c})+\frac{\upsilon }{G(\upsilon )\Gamma (\upsilon )}\int_{0}^{t}F_{2}(\rho ,I_{c}){\left(t-\rho \right)}^{\upsilon -1}d\rho ,X_{v}(t)-X_{v}(0)=\frac{1-\upsilon }{\Gamma (\upsilon )}F_{3}(t,X_{v})+\frac{\upsilon }{G(\upsilon )\Gamma (\upsilon )}\int_{0}^{t}F_{3}(\rho ,X_{v}){\left(t-\rho \right)}^{\upsilon -1}d\rho ,Y_{v}(t)-Y_{v}(0)=\frac{1-\upsilon }{\Gamma (\upsilon )}F_{4}(t,Y_{v})+\frac{\upsilon }{G(\upsilon )\Gamma (\upsilon )}\int_{0}^{t}F_{4}(\rho ,Y_{v}){\left(t-\rho \right)}^{\upsilon -1}d\rho .$$

Take into account the set S=M(I)×M(I)×M(I)×M(I) where M(I)=C[0,T] is the Banach space of real-valued continuous functions defined on an interval I=[0,T] with the corresponding norm defined by $\left\|S_{c},I_{c},X_{v},Y_{v}\|=\|S_{c}\|+\|I_{c}\|+\|X_{v}\|+\|Y_{v}\right\|$, where

$$\|S_{c}\|=\underset{t\in I}{\sup}|S_{c}(t)|,\|I_{c}\|=\underset{t\in I}{\sup}|I_{c}(t)|,\|X_{v}\|=\underset{t\in I}{\sup}|X_{v}(t)|,\|Y_{v}\|=\underset{t\in I}{\sup}|Y_{v}(t)|.$$

Theorem 2 *For any of the kernels*$F_{1},F_{2},F_{3},F_{4}$*in*(2)*, there exists*$V_{i}>0,i=1,\ldots ,4$*, such that*

(9) $$\|F_{1}(t,S_{c})-F_{1}(t,S_{c\;1})\|\leq V_{1}\|S_{c}-S_{c\;1}(t)\|,\|F_{2}(t,I_{c})-F_{2}(t,I_{c\;1})\|\leq V_{2}\|I_{c}-I_{c\;1}(t)\|,\|F_{3}(t,X_{v})-F_{3}(t,X_{v\;1})\|\leq V_{3}\|X_{v}-X_{v\;1}(t)\|,\|F_{4}(t,Y_{v})-F_{4}(t,Y_{v\;1})\|\leq V_{4}\|Y_{v}-Y_{v\;1}(t)\|,$$

*and are contractions for*$0\leq V_{i}<1,i=1,\ldots ,4$*.*

Proof $$\|F_{1}(t,S_{c})-F_{1}(t,S_{c\;1})\|=\|m_{1}-\alpha_{1}S_{c}Y_{v}-dS_{c}-\left(m_{1}-\alpha_{1}S_{c\;1}Y_{v}-dS_{c\;1}\right)\|=\|-\alpha_{1}S_{c}Y_{v}-dS_{c}+\alpha_{1}S_{c\;1}Y_{v}+dS_{c\;1}\|=\|\alpha_{1}Y_{v}(S_{c\;1}-S_{c})+d(S_{c\;1}-S_{c})\|\leq (\alpha_{1}n_{1}+d)\|(S_{c\;1}-S_{c})\|\leq V_{1}\|(S_{c\;1}-S_{c})\|,$$

where $V_{1}=\alpha_{1}n_{1}+d$.

$$\|S_{c}\|=\underset{\tau \in I}{\sup}|S_{c}(t)|=n_{4},\|I_{c}\|=\underset{\tau \in I}{\sup}|I_{c}(t)|=n_{3},\|X_{v}\|=\underset{\tau \in I}{\sup}|X_{v}(t)|=n_{2},\|Y_{v}\|=\underset{\tau \in I}{\sup}|Y_{v}(t)|=n_{1}.$$

As a result, $F_{1}(t,S_{c})$ fulfills the Lipschitz condition with Lipschitz constant $V_{1}=\alpha_{1}n_{1}+d$. Furthermore, if $0\leq V_{1}<1$, then $F_{1}(t,S_{c})$ is a contraction. Similarly, we can demonstrate the existence of $V_{i},i=2,3,4$, and a contraction principle for $F_{2}(t,I_{c}),F_{3}(t,X_{v}),F_{4}(t,Y_{v}),0\leq V_{i}<1$. Here, for $t=t_{n},n=1,2,\ldots$, define the recursive form of (8) shown below

(10) $$S_{c\;n}(t)=\frac{1-\upsilon }{G(\upsilon )}F_{1}(t,S_{c\;n-1})+\frac{\upsilon }{G(\upsilon )\Gamma (\upsilon )}\int_{0}^{t}F_{1}(t,S_{c\;n-1}){\left(t-\rho \right)}^{\upsilon -1}d\rho ,I_{c\;n}(t)=\frac{1-\upsilon }{G(\upsilon )}F_{2}(t,S_{c\;n-1})+\frac{\upsilon }{G(\upsilon )\Gamma (\upsilon )}\int_{0}^{t}F_{2}(t,I_{c\;n-1}){\left(t-\rho \right)}^{\upsilon -1}d\rho ,X_{v\;n}(t)=\frac{1-\upsilon }{G(\upsilon )}F_{3}(t,X_{v\;n-1})+\frac{\upsilon }{G(\upsilon )\Gamma (\upsilon )}\int_{0}^{t}F_{3}(t,X_{v\;n-1}){\left(t-\rho \right)}^{\upsilon -1}d\rho ,Y_{v\;n}(t)=\frac{1-\upsilon }{G(\upsilon )}F_{4}(t,Y_{v\;n-1})+\frac{\nu }{G(\upsilon )\Gamma (\upsilon )}\int_{0}^{t}F_{4}(t,Y_{v\;n-1}){\left(t-\rho \right)}^{\upsilon -1}d\rho ,$$

with initial conditions (3). The differences between successive terms are represented in (10) in the following manner:

$$H_{1n}(t)=S_{c\;n}(t)-S_{c\;n-1}(t),=\frac{1-\upsilon }{G(\upsilon )}\left(F_{1}(t,S_{c\;n-1})-F_{1}(t,S_{c\;n-2})\right)+\frac{\upsilon }{G(\upsilon )\Gamma (\upsilon )}\int_{0}^{t}\left(F_{1}(t,S_{c\;n-1})-F_{1}(t,S_{c\;n-2})\right){\left(t-\rho \right)}^{\upsilon -1}d\rho ,H_{2n}(t)=I_{c\;n}(t)-I_{c\;n-1}(t),=\frac{1-\upsilon }{G(\upsilon )}\left(F_{2}(t,I_{c\;n-1})-F_{2}(t,I_{c\;n-2})\right)+\frac{\upsilon }{G(\upsilon )\Gamma (\upsilon )}\int_{0}^{t}\left(F_{2}(t,I_{c\;n-1})-F_{2}(t,I_{c\;n-2})\right){\left(t-\rho \right)}^{\upsilon -1}d\rho ,H_{3n}(t)=S_{c\;n}(t)-X_{v\;n-1}(t),=\frac{1-\upsilon }{G(\upsilon )}\left(F_{3}(t,X_{v\;n-1})-F_{3}(t,X_{v\;n-2})\right)+\frac{\upsilon }{G(\upsilon )\Gamma (\upsilon )}\int_{0}^{t}\left(F_{3}(t,X_{v\;n-1})-F_{3}(t,X_{v\;n-2})\right){\left(t-\rho \right)}^{\upsilon -1}d\rho ,H_{4n}(t)=Y_{v\;n}(t)-Y_{v\;n-1}(t),=\frac{1-\upsilon }{G(\upsilon )}\left(F_{4}(t,Y_{v\;n-1})-F_{4}(t,Y_{v\;n-2})\right)+\frac{\upsilon }{G(\upsilon )\Gamma (\upsilon )}\int_{0}^{t}\left(F_{4}(t,Y_{v\;n-1})-F_{4}(t,Y_{v\;n-2})\right){\left(t-\rho \right)}^{\upsilon -1}d\rho .$$

In (6), we have the norm on both sides of each equation

(11) $$\|H_{1n}(t)\|=\|S_{c\;n}(t)-S_{c\;n-1}(t)\|,=\frac{1-\upsilon }{G(\upsilon )}\|F_{1}(t,S_{c\;n-1})-F_{1}(t,S_{c\;n-2})\|+\frac{\upsilon }{G(\upsilon )\Gamma (\upsilon )}\int_{0}^{t}\|F_{1}(t,S_{c\;n-1})-F_{1}(t,S_{c\;n-2})\|{\left(t-\rho \right)}^{\upsilon -1}d\rho ,\|H_{2n}(t)\|=\|I_{c\;n}(t)-I_{c\;n-1}(t)\|,=\frac{1-\upsilon }{G(\upsilon )}\|F_{2}(t,I_{c\;n-1})-F_{2}(t,I_{c\;n-2})\|+\frac{\upsilon }{G(\upsilon )\Gamma (\upsilon )}\int_{0}^{t}\|F_{2}(t,I_{c\;n-1})-F_{2}(t,I_{c\;n-2})\|{\left(t-\rho \right)}^{\upsilon -1}d\rho ,\|H_{3n}(t)\|=\|S_{c\;n}(t)-X_{v\;n-1}(t)\|=\frac{1-\upsilon }{G(\upsilon )}\|F_{3}(t,X_{v\;n-1})-F_{3}(t,X_{v\;n-2})\|+\frac{\upsilon }{G(\upsilon )\Gamma (\upsilon )}\int_{0}^{t}\|F_{3}(t,X_{v\;n-1})-F_{3}(t,X_{v\;n-2})\|{\left(t-\rho \right)}^{\upsilon -1}d\rho ,\|H_{4n}(t)\|=\|Y_{v\;n}(t)-Y_{v\;n-1}(t)\|,=\frac{1-\upsilon }{G(\upsilon )}\|F_{4}(t,Y_{v\;n-1})-F_{4}(t,Y_{v\;n-2})\|+\frac{\upsilon }{G(\upsilon )\Gamma (\upsilon )}\int_{0}^{t}\|F_{4}(t,Y_{v\;n-1})-F_{4}(t,Y_{v\;n-2})\|{\left(t-\rho \right)}^{\upsilon -1}d\rho .$$

Moreover, the first equality in (11) can be simplified as follows:

$$\|H_{1n}(t)\|=\|S_{c\;n}(t)-S_{c\;n-1}(t)\|,\leq \frac{1-\upsilon }{G(\upsilon )}\|F_{1}(t,S_{c\;n-1})-F_{1}(t,S_{c\;n-2})\|+\frac{\upsilon }{G(\upsilon )\Gamma (\upsilon )}\int_{0}^{\tau }\|F_{1}(t,S_{c\;n-1})-F_{1}(t,S_{c\;n-2})\|{\left(t-\rho \right)}^{\nu -1}d\rho ,\leq \frac{1-\upsilon }{G(\upsilon )}V_{1}\|S_{c\;n-1})-S_{c\;n-2})\|+\frac{\upsilon }{G(\upsilon )\Gamma (\upsilon )}\int_{0}^{\tau }\|S_{c\;n-1})-S_{c\;n-2})\|{\left(t-\rho \right)}^{\upsilon -1}d\rho ,\leq V_{1}\|H_{1(n-1)}(t)\|\left|\frac{1-\upsilon }{G(\upsilon )}+\frac{t^{\upsilon }}{G(\upsilon )\Gamma (\upsilon )}\right|.$$

As a consequence, we now have

(12) $$\|H_{1n}(t)\|\leq V_{1}\left|\frac{1-\upsilon }{G(\upsilon )}+\frac{t^{\upsilon }}{G(\upsilon )\Gamma (\upsilon )}\right|\|H_{1(n-1)}(t)\|.$$

Similarly, the leftover expressions of (4) can be simplified to the following:

(13) $$\|H_{2n}(t)\|\leq V_{2}\left|\frac{1-\upsilon }{G(\upsilon )}+\frac{t^{\upsilon }}{G(\upsilon )\Gamma (\upsilon )}\right|\|H_{2(n-1)}(t)\|,\|H_{3n}(t)\|\leq V_{3}\left|\frac{1-\upsilon }{G(\upsilon )}+\frac{t^{\upsilon }}{G(\upsilon \Gamma (\upsilon )}\right|\|H_{3(n-1)}(t)\|,\|H_{4n}(t)\|\leq V_{4}\left|\frac{1-\upsilon }{G(\upsilon )}+\frac{t^{\upsilon }}{G(\upsilon )\Gamma (\upsilon )}\right|\|H_{4(n-1)}(t)\|.$$

 □

Theorem 3 *The ABC fractional model given in* (2) *has a solution if we determine* $\Pi_{0}^{\upsilon }$ *such that it satisfies the inequality.*

$$\left(\frac{1-\upsilon }{G(\upsilon )}+\frac{\Pi_{0}^{\upsilon }}{G(\upsilon )\Gamma (\upsilon )}\right)V_{i}<1,i=1,2,3,4.$$

Proof From (12) and (13) we get

$$\|H_{1n}(t)\|\leq \|S_{c}(0)\|{\left[\left(\frac{1-\upsilon }{G(\upsilon )}+\frac{\Pi_{0}^{\upsilon }}{G(\upsilon )\Gamma (\upsilon )}\right)V_{1}\right]}^{n},\|H_{2n}(t)\|\leq \|I_{c}(0)\|{\left[\left(\frac{1-\upsilon }{G(\upsilon )}+\frac{\Pi_{0}^{\upsilon }}{G(\upsilon )\Gamma (\upsilon )}\right)V_{2}\right]}^{n},\|H_{3n}(t)\|\leq \|X_{v}(0)\|{\left[\left(\frac{1-\upsilon }{G(\upsilon )}+\frac{\Pi_{0}^{\upsilon }}{G(\upsilon )\Gamma (\upsilon )}\right)V_{3}\right]}^{n},\|H_{4n}(t)\|\leq \|Y_{v}(0)\|{\left[\left(\frac{1-\upsilon }{G(\upsilon )}+\frac{\Pi_{0}^{\upsilon }}{G(\upsilon )\Gamma (\upsilon )}\right)V_{4}\right]}^{n}.$$

Theorem 2 indicates that there is a fixed point, so must now demonstrate that the functions $S_{c}(t),I_{c}(t),X_{v}(t),Y_{v}(t)$ are solutions (2). Suppose the following circumstances are valid:

(14) $$S_{c}(t)-S_{c}(0)=S_{c\;n}(t)-h_{1n}(t),I_{c}(t)-I_{c}(0)=I_{c\;n}(t)-h_{2n}(t),X_{v}(t)-X_{v}(0)=X_{v\;n}(t)-h_{3n}(t),Y_{v}(t)-Y_{v}(0)=Y_{v\;n}(t)-h_{4n}(t).$$

From (14) we get

$$\|h_{1n}(t)\|\leq \frac{1-\upsilon }{G(\upsilon )}\|\left(F_{1}(\tau ,S_{c\;n})-F_{1}(\tau ,S_{c\;n-1})\right)\|{\left(\tau -\rho \right)}^{\upsilon -1}d\rho ,\leq \frac{1-\upsilon }{G(\upsilon )}V_{1}\|S_{c\;n}-S_{c\;n-1}\|+\frac{\upsilon^{n}}{G(\upsilon )\Gamma (\upsilon )}V_{1}\|S_{c\;n}-S_{c\;n-1}\|.$$

Recursion of the process results in

$$\|h_{1n}(t)\|\leq {\left[\frac{1-\upsilon }{G(\upsilon )}+\frac{t^{\upsilon }}{G(\upsilon )\Gamma (\upsilon )}\right]}^{n+1}V_{1}^{n}{\left\|S_{c\;n}-S_{c\;n-1}\right\|}^{n}$$

which at $t=\Pi_{0}^{\upsilon }$ gives

(15) $$\|h_{1n}(t)\|\leq {\left[\frac{1-\upsilon }{G(\upsilon )}+\frac{\Pi_{0}^{\upsilon }}{G(\upsilon )\Gamma (\upsilon )}\right]}^{n+1}V_{1}^{n}{\left\|S_{c\;n}-S_{c\;n-1}\right\|}^{n},\|h_{1n}(t)\|\to 0.$$

Taking the limit and applying on (15) as n→∞, we see that $\|h_{1n}(t)\|\to 0$ for

$$\left[\frac{1-\upsilon }{G(\upsilon )}+\frac{t^{\upsilon }}{G(\upsilon )\Gamma (\upsilon )}\right]V_{1}<1.$$

Likewise, we can demonstrate that $\|h_{2n}(t)\|\to 0,\|h_{3n}(t)\|\to 0,\|h_{4n}(t)\|\to 0$,

$$\left[\frac{1-\upsilon }{G(\upsilon )}+\frac{t^{\upsilon }}{G(\upsilon )\Gamma (\upsilon )}\right]V_{i}<1,\;i=2,3,4.$$

The Banach fixed point theorem guarantees the existence of the solution of model (2). □

Theorem 4 **Uniqueness of solution**

*The AB fractional model* (2) *has a unique solution if*

$$\left[\frac{1-\upsilon }{G(\upsilon )}+\frac{t^{\upsilon }}{G(\upsilon )\Gamma (\upsilon )}\right]V_{i}<1,\;i=1,\ldots 4.$$

Proof Let us suppose that $S_{c\;1}(t),I_{c\;1}(t),X_{v\;1}(t),Y_{v\;1}(t)$ are also solutions to (2). Then

$$S_{c}(t)-S_{c\;1}(t)=\frac{1-\upsilon }{G(\upsilon )}\left(F_{1}(t,S_{c})-F_{1}(t,S_{c\;1})\right)+\frac{\upsilon }{G(\upsilon )\Gamma (\upsilon )}\int_{0}^{t}\left(F_{1}(t,S_{c})-F_{1}(t,S_{c\;1})\right){\left(t-\rho \right)}^{\upsilon -1}d\rho .$$

Using both sides' norms, we get

$$\|S_{c}(t)-S_{c\;1}(t)\|=\frac{1-\upsilon }{G(\upsilon )}V_{1}\|S_{c}-S_{c\;1}\|+\frac{t^{\upsilon }}{G(\upsilon )\Gamma (\upsilon )}V_{1}\|S_{c}-S_{c\;1}\|.$$

Since $\left(1-V_{1}\left(\frac{1-\upsilon }{G(\upsilon )}+\frac{t^{\upsilon }}{G(\upsilon )\Gamma (\upsilon )}\right)\right)>0$, we obtain $\|S_{c}(t)-S_{c\;1}(t)\|=0$. Hence, we get $S_{c}(t)=S_{c\;1}(t)$. Likewise, we can demonstrate that $I_{c}(t)=I_{c\;1}(t),X_{v}(t)=X_{v\;1}(t),Y_{v}=Y_{v\;1}(t)$. □

## 5 Hyers-Ulam (HU) stability

This part evaluates the stability of the fractional model. Different stability types have been used to analyze the epidemic model. Because HU-type stability has the advantage of providing an approximative solution when a problem is complex, many researchers have recently used it for epidemic models. [43] and [44] for more information about the HU stability. In this analysis, we use HU-type stability for CLCuV. For quick proof, we first demonstrate that model (2) is HU stable.

Definition 3 The CLCuV model is HU stable if for $\omega_{i}>0,L_{i}>0,i=1,2,3,4$ and for all $(S_{c}^{⁎},I_{c}^{⁎},X_{v}^{⁎},Y_{v}^{⁎})\in N$, satisfying below inequality

(16) $$|S_{c}^{⁎}-F_{1}(t,S_{c}(t))|\leq \omega_{1},|I_{c}^{⁎}-F_{2}(t,I_{c}(t))|\leq \omega_{2},|X_{v}^{⁎}-F_{3}(t,X_{v}(t))|\leq \omega_{3},|Y_{v}^{⁎}-F_{4}(t,Y_{v}(t))|\leq \omega_{4},$$

such that for all $(S_{c},I_{c},X_{v},Y_{v})\in N$ satisfying model (2) with below inequality

$$|S_{c}^{⁎}-S_{c}|\leq \omega_{1}L_{1},|I_{c}^{⁎}-I_{c}|\leq \omega_{2}L_{2},|X_{v}^{⁎}-X_{v}|\leq \omega_{3}L_{4},|Y_{v}^{⁎}-Y_{v}|\leq \omega_{4}L_{4}.$$

Remark 1 The $(S_{c}^{⁎},I_{c}^{⁎},X_{v}^{⁎},Y_{v}^{⁎})\in N$ is a solution of (16) if and only if there exists $K_{i}\in C([0,\theta ],R)$ for all t∈[0,θ], then, $|K_{i}(t)|<\omega_{i}$ and

$$S_{c}^{⁎}(t)=F_{1}(t,S_{c}(t))+K_{1}(t),I_{c}^{⁎}(t)=F_{2}(t,I_{c}(t))+K_{2}(t),X_{v}^{⁎}(t)=F_{3}(t,X_{v}(t))+K_{3}(t),Y_{v}^{⁎}(t)=F_{4}(t,Y_{v}(t))+K_{4}(t).$$

Lemma 4 *Let* $(S_{c}^{⁎},I_{c}^{⁎},X_{v}^{⁎},Y_{v}^{⁎})\in N$ *be a solution of* (16) *and for all* $\omega_{i}>0$ *. Then, the function* $(S_{c}^{⁎},I_{c}^{⁎},X_{v}^{⁎},Y_{v}^{⁎})\in \text{F}$ *satisfies*

(17) $$|S_{c}^{⁎}(t)-\left(S_{c}(0)+\frac{\left(1-\upsilon \right)}{G(\upsilon )}F_{1}(t,S_{c}(t))+\frac{\upsilon }{G(\upsilon )\Gamma (\upsilon )}\int_{0}^{t}{\left(t-\delta \right)}^{\upsilon -1}F_{1}(\delta ,S_{c}(\delta ))d\delta \right)|\leq \left[\frac{\left(1-\upsilon \right)}{G(\theta^{\upsilon })}+\frac{\upsilon }{G(\upsilon )\Gamma (\upsilon )}\right]\omega_{1},$$

(18) $$|I_{c}^{⁎}(t)-\left(I_{c}(0)+\frac{\left(1-\upsilon \right)}{G(\upsilon )}F_{2}(t,I_{c}(t))+\frac{\upsilon }{G(\upsilon )\Gamma (\upsilon )}\int_{0}^{t}{\left(t-\delta \right)}^{\upsilon -1}F_{2}(\delta ,I_{c}(\delta ))d\delta \right)|\leq \left[\frac{\left(1-\upsilon \right)}{G(\theta^{\upsilon })}+\frac{\upsilon }{G(\upsilon )\Gamma (\upsilon )}\right]\omega_{2},$$

(19) $$|X_{v}^{⁎}(t)-\left(X_{v}(0)+\frac{\left(1-\upsilon \right)}{G(\upsilon )}F_{3}(t,X_{v}(t))+\frac{\upsilon }{G(\upsilon )\Gamma (\upsilon )}\int_{0}^{t}{\left(t-\delta \right)}^{\upsilon -1}F_{3}(\delta ,X_{v}(\delta ))d\delta \right)|\leq \left[\frac{\left(1-\upsilon \right)}{G(\theta^{\upsilon })}+\frac{\upsilon }{G(\upsilon )\Gamma (\upsilon )}\right]\omega_{3},$$

(20) $$|Y_{v}^{⁎}(t)-\left(Y_{v}(0)+\frac{\left(1-\upsilon \right)}{G(\upsilon )}F_{4}(t,I_{c}(t))+\frac{\upsilon }{G(\upsilon )\Gamma (\upsilon )}\int_{0}^{t}{\left(t-\delta \right)}^{\upsilon -1}F_{4}(\delta ,Y_{v}(\delta ))d\delta \right)|\leq \left[\frac{\left(1-\upsilon \right)}{G(\theta^{\upsilon })}+\frac{\upsilon }{G(\upsilon )\Gamma (\upsilon )}\right]\omega_{4},$$

Proof Let $\omega_{i}>0$ and since we know $S^{⁎}\in F$ holds $S^{⁎}-F_{1}(t,S_{c}(t))\leq \omega_{1}$ and by Remark 1, we have $S_{c}^{⁎}(t)=F_{1}(t,S_{c}(t))+K_{1}(t)$ as $|K_{1}(t)|\leq \omega_{1}$. Then it follows that

$$S_{c}^{⁎}(t)=S_{c}{\left(0\right)}^{⁎}+\frac{\left(1-\upsilon \right)}{G(\upsilon )}[F_{1}(t,S_{c}(t))+K_{1}(t)]+\frac{\upsilon }{G(\nu )\Gamma (\upsilon )}\int_{0}^{t}{\left(t-\delta \right)}^{\upsilon -1}[F_{1}(\delta ,S_{c}(\delta ))+K_{1}(t)]d\delta .$$

Then, we have

$$|S_{c}^{⁎}(t)-\left(S_{c}(0)+\frac{\left(1-\upsilon \right)}{G(\upsilon )}F_{1}(t,S_{c}(t))+\frac{\upsilon }{G(\upsilon )\Gamma (\upsilon )}\int_{0}^{t}{\left(t-\delta \right)}^{\upsilon -1}F_{1}(\delta ,S_{c}(\delta ))d\delta \right)|\leq \frac{\left(1-\upsilon \right)}{G(\upsilon )}|K_{1}(t)|+\frac{\upsilon }{G(\upsilon )\Gamma (\upsilon )}\int_{0}^{t}{\left(t-\delta \right)}^{\upsilon -1}|K_{1}(t)|d\delta ,=|\frac{\left(1-\upsilon \right)}{G(\theta^{\upsilon })}+\frac{\upsilon }{G(\nu )\Gamma (\upsilon )}|\omega_{1}.$$

This establishes inequality (17). Likewise, inequalities (18)–(20) are attainable. Our proposed model (2) is now proven to be HU-stable. □

Theorem 5 *Suppose that* (9) *, then our propose model* (2) *is HU-stable such that*

$$\left[\frac{\left(1-\upsilon \right)}{G(\upsilon )}+\frac{\theta^{\upsilon }}{G(\upsilon )\Gamma (\upsilon )}\right]A_{i}<1,$$

*where* $A_{i},i=1,\ldots 4$ *is a Lipschitz constant.*

Proof Since we are aware that $S_{c}(t)\in F$ holds for (16), let $\omega_{i}>0$. Additionally, using Theorem 4, we assume that $S_{c}(t)inF$ is the only solution to the suggested model (2). Thus,

(21) $$S_{c}(t)=S_{c}(0)+\frac{\left(1-\upsilon \right)}{G(\upsilon )}F_{1}(t,S_{c}(t))+\frac{\upsilon }{G(\upsilon )\Gamma (\upsilon )}\int_{0}^{t}{\left(t-\delta \right)}^{\upsilon -1}F_{1}(\delta ,S_{c}(\delta ))d\delta .$$

Consequently, we calculate using Lemma 4 and the properties of the triangular inequality to (21).

$$|S_{c}^{⁎}(t)-S_{c}(t)|=|S_{c}{\left(0\right)}^{⁎}-S_{c}(0)-\frac{\left(1-\upsilon \right)}{G(\upsilon )}F_{1}(t,S_{c}(t))-\frac{\upsilon }{G(\upsilon )\Gamma (\upsilon )}\int_{0}^{t}{\left(t-\delta \right)}^{\upsilon -1}F_{1}(\delta ,S_{c}(\delta ))d\delta |\leq |S_{c}^{⁎}(t)-\left(S_{c}(0)+\frac{\left(1-\upsilon \right)}{G(\upsilon }F_{1}(t,S_{c}(t))+\frac{\upsilon }{G(\upsilon )\Gamma (\upsilon )}\int_{0}^{t}{\left(t-\delta \right)}^{\upsilon -1}F_{1}(t,S_{c}(\delta ))d\delta \right)|+\frac{\left(1-\upsilon \right)}{G(\upsilon )}|F_{1}(t,S_{c}(t))-F_{1}(t,S_{c}^{⁎}(t))|+\frac{\upsilon }{G(\upsilon )\Gamma (\upsilon )}\int_{0}^{t}{\left(t-\delta \right)}^{\upsilon -1}|F_{1}(\delta ,S_{c}(\delta ))-F_{1}(\delta ,S_{c}^{⁎}(\delta ))|d\delta \leq \left[\frac{\left(1-\upsilon \right)}{G(\theta^{\upsilon })}+\frac{\upsilon }{G(\upsilon )\Gamma (\upsilon )}\right]\omega_{1}+\left[\frac{\left(1-\upsilon \right)}{G(\upsilon )}+\frac{\theta^{\upsilon }}{G(\upsilon )\Gamma (\upsilon )}\right]A_{1}||S_{c}^{⁎}-S_{c}||.$$

Hence,

$$|S_{c}^{⁎}(t)-S_{c}(t)|\leq \frac{\left[\frac{\left(1-\upsilon \right)}{G(\theta^{\upsilon })}+\frac{\upsilon }{G(\upsilon )\Gamma (\upsilon )}\right]\omega_{1}}{1-\left[\frac{\left(1-\upsilon \right)}{G(\upsilon )}+\frac{\theta^{\upsilon }}{G(\upsilon )\Gamma (\upsilon )}\right]A_{1}}.$$

We let $L_{1}=|S_{c}^{⁎}(t)-S_{c}(t)|\leq \frac{\left[\frac{\left(1-\upsilon \right)}{G(\theta^{\upsilon })}+\frac{\upsilon }{G(\upsilon )\Gamma (\upsilon )}\right]\omega_{1}}{1-\left[\frac{\left(1-\upsilon \right)}{G(\upsilon )}+\frac{\theta^{\upsilon }}{G(\upsilon )\Gamma (\upsilon )}\right]A_{1}}$, then $|S_{c}^{⁎}-S_{c}|\leq \omega_{1}L_{1}$. Similarly,

$$|I_{c}^{⁎}-I_{c}|\leq \omega_{2}L_{2},|X_{v}^{⁎}-X_{v}|\leq \omega_{3}L_{3},Y_{v}^{⁎}-Y_{v}|\leq \omega_{4}L_{4}.$$

Hence, the system model equation (2) is HU-stable. □

## 6 Numerical schemes of the model

Here, we determine a numerical scheme for model (2) based on the Toufik Atangana rule discussed in [45]. The first equation of (2) gives us the following.

(22) Dtυ0ABCSc=F1(t,Sc(t)),Sc(0)=Sc0.

Using the information from (8), we get the solution for (22) in (23):

(23) $$S_{c}(t)=S_{c}(0)+\frac{1-\upsilon }{G(\upsilon )}F_{1}(t,S_{c}(t))+\frac{\upsilon }{G(\upsilon )\Gamma (\upsilon )}\int_{0}^{t}F_{1}(\rho ,S_{c}(\rho )){\left(t-\rho \right)}^{\upsilon -1}d\rho .$$

Using Lagrange's interpolation polynomial on the interval $\left[t_{k},t_{k+1}\right]$ to the equality $F_{1}(w,S_{c}(w))=m_{1}-\alpha_{1}S_{c}(w)Y_{v}(w)-dS_{c}(w)$ leads to

(24) $$S_{c}k\approx \frac{1}{h}\left[(w-t_{k-1})F_{1}(t_{k},S_{c}(t_{k}),I_{c}(t_{k}),X_{v}(t_{k}),Y_{v}(t_{k}))\right]-\frac{1}{h}\left[(w-t_{k})F_{1}(t_{k-1},S_{c}(t_{k-1}),I_{c}(t_{k-1}),X_{v}(t_{k-1}),Y_{v}(t_{k-1}))\right],$$

where $h=t_{k}-t_{k-1}$. Equation (24) is introduced into equation (23) to produce

(25) $$S_{c}(t_{s+1})=S_{c}(0)+\frac{1-\upsilon }{G(\upsilon )}F_{1}(t_{k},S_{c}(t_{k}),I_{c}(t_{k}),X_{v}(t_{k}),Y_{v}(t_{k}))+\frac{\upsilon }{G(\upsilon )\Gamma (\upsilon )}\sum_{j=1}^{s}\frac{F_{1}(t_{j},S_{c}(t_{j}),I_{c}(t_{j}),X_{v}(t_{j}),Y_{v}(t_{j}))}{h}\int_{t_{j}}^{t_{j+1}}(w-t_{j-1}){\left(t_{s+1}-w\right)}^{\upsilon -1}dw+\frac{\upsilon }{G(\upsilon )\Gamma (\upsilon )}\sum_{j=1}^{s}-\frac{F_{1}(t_{j-1},S_{c}(t_{j-1}),I_{c}(t_{j-1}),X_{v}(t_{j-1}),Y_{v}(t_{j-1}))}{h}\int_{t_{j}}^{t_{j+1}}(w-t_{j-1}){\left(t_{s+1}-w\right)}^{\upsilon -1}dw,=S_{c}(0)+\frac{1-\upsilon }{G(\upsilon )}F_{1}(t_{s},S_{c}(t_{s}),I_{c}(t_{s}),X_{v}(t_{s}),Y_{v}(t_{s}))+\frac{\upsilon }{G(\upsilon )\Gamma (\upsilon )}\sum_{j=1}^{s}\frac{F_{1}(t_{j},S_{c}(t_{j}),I_{c}(t_{j}),X_{v}(t_{j}),Y_{v}(t_{j}))}{h}M_{j-1}+G(\upsilon )\Gamma (\upsilon )\sum_{j=1}^{s}-\frac{F_{1}(t_{j-1},S_{c}(t_{j-1}),I_{c}(t_{j-1}),X_{v}(t_{j-1}),Y_{v}(t_{j-1}))}{h}M_{j},$$

where

(26) $$M_{j-1}=\int_{t_{j}}^{t_{j}+1}(w-t_{j-1}){\left(t_{s+1}-w\right)}^{\upsilon -1}dw=-\frac{1}{\upsilon }\left[(t_{j+1}-t_{j-1}){\left(t_{s+1}-t_{j+1}\right)}^{\upsilon }-(t_{j}-t_{j-1}){\left(t_{s+1}-t_{j}\right)}^{\upsilon }\right]-\frac{1}{\upsilon (\upsilon +1)}\left[{\left(t_{s+1}-t_{j+1}\right)}^{\upsilon +1}{\left(t_{s+1}-t_{j+1}\right)}^{\upsilon }-{\left(t_{s+1}-t_{j}\right)}^{\upsilon +1}\right],$$

(27) $$M_{j}=\int_{t_{j}}^{t_{j}+1}(w-t_{j-1}){\left(t_{s+1}-w\right)}^{\upsilon -1}dw=-\frac{1}{\upsilon }\left[(t_{j+1}-t_{j-1}){\left(t_{s+1}-t_{j+1}\right)}^{\upsilon }\right]-\frac{1}{\upsilon (\upsilon +1)}\left[{\left(t_{s+1}-t_{j+1}\right)}^{\upsilon +1}-{\left(t_{s+1}-t_{j}\right)}^{\upsilon +1}\right].$$

Furthermore, plugging $t_{j}=jh$ into equation (26) and (27) yields

(28) $$M_{j-1}=\frac{h^{\upsilon +1}}{\upsilon (\upsilon +1)}\left[{\left(s+1-j\right)}^{\upsilon }(s-j+2+\upsilon )-{\left(s-j\right)}^{\upsilon }(s-j+2+2\upsilon )\right],$$

(29) $$M_{j}=\frac{h^{\upsilon +1}}{\upsilon (\upsilon +1)}\left[{\left(s+1-j\right)}^{\upsilon +1}-{\left(s-j\right)}^{\upsilon }(s-j+1+\upsilon )\right].$$

Finally, the expression of equation (25) in terms of equations (28) and (29) is as follows:

(30) $$S_{c}(t_{s+1})=S_{c}(t_{0})+\frac{1-\upsilon }{G(\upsilon )}F_{1}(t_{s},S_{c}(t_{s}),I_{c}(t_{s}),X_{v}(t_{s}),Y_{v}(t_{s}))+\frac{\upsilon }{G(\upsilon )\Gamma (\upsilon )}\times \sum_{j=1}^{s}\frac{F_{1}(t_{j},S_{c}(t_{j}),I_{c}(t_{j}),X_{v}(t_{j}),Y_{v}(t_{j}))}{\Gamma (\upsilon +2)}\times h^{\upsilon }\left[{\left(s+1-j\right)}^{\upsilon }(s-j+2+\upsilon )-{\left(s-j\right)}^{\upsilon }(s-j+2+2\upsilon )\right]+\frac{\upsilon }{G(\upsilon )\Gamma (\upsilon )}\times \sum_{j=1}^{s}-\frac{F_{1}(t_{j-1},S_{c}(t_{j-1}),I_{c}(t_{j-1}),X_{v}(t_{j-1}),Y_{v}(t_{j-1}))}{\Gamma (\upsilon +2)}\times h^{\upsilon }\left[{\left(s+1-j\right)}^{\upsilon +1}-{\left(s-j\right)}^{\upsilon }(s-j+1+\upsilon )\right].$$

Similarly, for the remaining state variables, there are the subsequent equations:

(31) $$I_{c}(t_{s+1})=I_{c}(t_{0})+\frac{1-\upsilon }{G(\upsilon )}F_{2}(t_{s},S_{c}(t_{s}),I_{c}(t_{s}),X_{v}(t_{s}),Y_{v}(t_{s}))+\frac{\upsilon }{G(\upsilon )\Gamma (\upsilon )}\times \sum_{j=1}^{s}\frac{F_{2}(t_{j},S_{c}(t_{j}),I_{c}(t_{j}),X_{v}(t_{j}),Y_{v}(t_{j}))}{\Gamma (\upsilon +2)}\times h^{\upsilon }\left[{\left(s+1-j\right)}^{\upsilon }(s-j+2+\upsilon )-{\left(s-j\right)}^{\upsilon }(s-j+2+2\upsilon )\right]+\frac{\upsilon }{G(\upsilon )\Gamma (\upsilon )}\times \sum_{j=1}^{s}-\frac{F_{2}(t_{j-1},S_{c}(t_{j-1}),I_{c}(t_{j-1}),X_{v}(t_{j-1}),Y_{v}(t_{j-1}))}{\Gamma (\upsilon +2)}\times h^{\upsilon }\left[{\left(s+1-j\right)}^{\upsilon +1}-{\left(s-j\right)}^{\upsilon }(s-j+1+\upsilon )\right].$$

(32) $$X_{v}(t_{s+1})=X_{v}(t_{0})+\frac{1-\upsilon }{G(\upsilon )}F_{3}(t_{s},S_{c}(t_{s}),I_{c}(t_{s}),X_{v}(t_{s}),Y_{v}(t_{s}))+\frac{\upsilon }{G(\upsilon )\Gamma (\upsilon )}\times \sum_{j=1}^{s}\frac{F_{3}(t_{j},S_{c}(t_{j}),I_{c}(t_{j}),X_{v}(t_{j}),Y_{v}(t_{j}))}{\Gamma (\upsilon +2)}\times h^{\upsilon }\left[{\left(s+1-j\right)}^{\upsilon }(s-j+2+\upsilon )-{\left(s-j\right)}^{\upsilon }(s-j+2+2\upsilon )\right]+\frac{\upsilon }{G(\upsilon )\Gamma (\upsilon )}\times \sum_{j=1}^{s}-\frac{F_{3}(t_{j-1},S_{c}(t_{j-1}),I_{c}(t_{j-1}),X_{v}(t_{j-1}),Y_{v}(t_{j-1}))}{\Gamma (\upsilon +2)}\times h^{\upsilon }\left[{\left(s+1-j\right)}^{\upsilon +1}-{\left(s-j\right)}^{\upsilon }(s-j+1+\upsilon )\right].$$

(33) $$Y_{v}(t_{s+1})=Y_{v}(t_{0})+\frac{1-\upsilon }{G(\upsilon )}F_{4}(t_{s},S_{c}(t_{s}),I_{c}(t_{s}),X_{v}(t_{s}),Y_{v}(t_{s}))+\frac{\upsilon }{G(\upsilon )\Gamma (\upsilon )}\times \sum_{j=1}^{s}\frac{F_{4}(t_{j},S_{c}(t_{j}),I_{c}(t_{j}),X_{v}(t_{j}),Y_{v}(t_{j}))}{\Gamma (\upsilon +2)}\times h^{\upsilon }\left[{\left(s+1-j\right)}^{\upsilon }(s-j+2+\upsilon )-{\left(s-j\right)}^{\upsilon }(s-j+2+2\upsilon )\right]+\frac{\upsilon }{G(\upsilon )\Gamma (\upsilon )}\times \sum_{j=1}^{s}-\frac{F_{4}(t_{j-1},S_{c}(t_{j-1}),I_{c}(t_{j-1}),X_{v}(t_{j-1}),Y_{v}(t_{j-1}))}{\Gamma (\upsilon +2)}\times h^{\upsilon }\left[{\left(s+1-j\right)}^{\upsilon +1}-{\left(s-j\right)}^{\upsilon }(s-j+1+\upsilon )\right].$$

## 7 Numerical simulation

**Figure 2:**
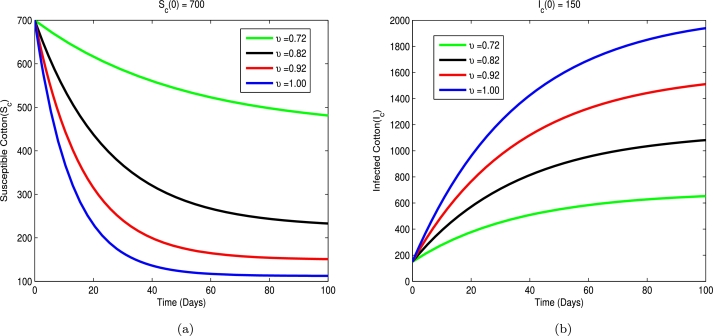
Total number of susceptible and infected plant with different values of *υ*.

**Figure 3:**
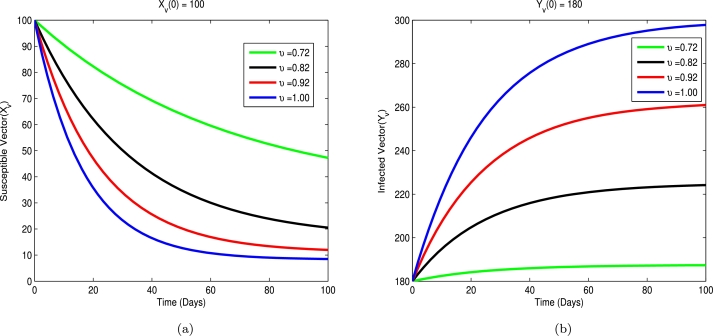
Total number of susceptible and infected vector with different values of *υ*.

In this part, the effects of different fractional order values on the model are explored. To this end, we present various results of the model using a numerical method created by Toufic and Antagana, as shown in equations (30)-(33). Following initial conditions are applied during simulations and analysis: $S_{c}(0)=700,I_{c}(0)=150,X_{v}(0)=100,Y_{v}(0)=180$, and the parameter values are given in Table 1. Fig. 2a shows that increasing the fractional order (*υ*), from 0.72 to 1.00 leads to decreasing number susceptible cotton plants $\left(S_{c}\right)$. Fig. 2b shows that increasing the fractional order (*υ*), from 0.72 to 1.00 leads to increasing number of infected plants $\left(I_{c}\right)$. Fig. 3a shows that increasing the fractional order (*υ*), from 0.72 to 1.00 leads to decreasing number susceptible vector population $\left(X_{v}\right)$. Fig. 3b shows that increasing the fractional order (*υ*), from 0.72 to 1.00 leads to increasing number of infected vector population $\left(Y_{v}\right)$.

**Table 1:** The parameter values.

Parameter	Value *day*^−1^	source
*m*_1_	0.700	Estimated
*m*_2_	0.270	[17]
*α*_1_	0.0004	Estimated
*α*_2_	0.0005	Estimated
*d*	0.040	[46]
*β*_1_	0.002	Estimated
*β*_2_	0.0024	Estimated
*ψ*	0.030	[17]

In other words, based on Figure 2, Figure 3, we can conclude that decreasing *υ*, significantly reduces the number of $I_{c}$ and $Y_{v}$ cases. Besides that, as shown in the figures, the curves for each compartment $I_{c}$, and $Y_{v}$ compress as decrease from 1.00 to 0.72. We can conclude that as gets closer to zero from the right, diseases in $I_{c}$, and $Y_{v}$ decreases.

This actually means that the memory impact of the dynamic system is larger when the derivative order is decreased from 1.00 to 0.72, and the infection in each compartment progressively increases for a long time. According to the data, crop infection growth is greatly decreased when the derivative's fractional order is decreased, and no change in rate of change is observed when the derivative's fractional order is decreased to zero. If there is no rate of change in the disease, the crop will be fruitful. In this case, the fractional order will be higher than 0. The derivative order also accounts for the importance of farmer expertise or historical knowledge of the disease. The rough results show that fractional order derivatives offer a lot of dynamics and frequently express biological systems more effectively.

## 8 Conclusion

An eco-epidemiological mathematical model that makes use of the fractional derivative of the Atangana Baleanu Caputo (ABC) has been studied for CLCuV dynamics. The model's stability study was done using the Hyers-Ulam approach, and it was proven that the solution existed and was distinct. The simulation results demonstrate that as *ϕ* increases from 0.72 to 1.00, the susceptible cotton $\left(S_{c}\right)$ and susceptible vector $\left(S_{v}\right)$ decreased. The graphs for infected cotton $\left(I_{c}\right)$ and infected vector $\left(Y_{v}\right)$ demonstrate that the disease spread gradually as the fractional order increased from 0.72 to 1.00. We assert that mathematical models involving the Atangana Baleanu Caputo (ABC) fractional operator can reveal more about the hidden or actual properties of real-world phenomena. We show graphical results with various values of *υ* and observed that as the order is decreased, the number of cases decreases. In a subsequent study, the effects of other fractional operators, such as Caputo-Fabrizio fractional derivatives, as well as optimal control and cost effectiveness, would be taken into account.

## Author contribution statement

Abayneh Kebede Fantaye, Zerihun Kinfe Birhanu: Conceived and designed the experiments; Performed the experiments; Analyzed and interpreted the data; Contributed reagents, materials, analysis tools or data; Wrote the paper.

## Declaration of Competing Interest

The authors declare that they have no known competing financial interests or personal relationships that could have appeared to influence the work reported in this paper.

## Data Availability

No data was used for the research described in the article.
